# Interaction of silver nanoparticles with HIV-1

**DOI:** 10.1186/1477-3155-3-6

**Published:** 2005-06-29

**Authors:** Jose Luis Elechiguerra, Justin L Burt, Jose R Morones, Alejandra Camacho-Bragado, Xiaoxia Gao, Humberto H Lara, Miguel Jose Yacaman

**Affiliations:** 1 Department of Chemical Engineering, The University of Texas at Austin, Austin, Texas 78712, USA; 2 Texas Materials Institute, The University of Texas at Austin, Austin, Texas 78712, USA; 3 Facultad de Ciencias Biológicas, Universidad Autónoma de Nuevo León, San Nicolás de los Garza, Nuevo León, Mexico

## Abstract

The interaction of nanoparticles with biomolecules and microorganisms is an expanding field of research. Within this field, an area that has been largely unexplored is the interaction of metal nanoparticles with viruses. In this work, we demonstrate that silver nanoparticles undergo a size-dependent interaction with HIV-1, with nanoparticles exclusively in the range of 1–10 nm attached to the virus. The regular spatial arrangement of the attached nanoparticles, the center-to-center distance between nanoparticles, and the fact that the exposed sulfur-bearing residues of the glycoprotein knobs would be attractive sites for nanoparticle interaction suggest that silver nanoparticles interact with the HIV-1 virus via preferential binding to the gp120 glycoprotein knobs. Due to this interaction, silver nanoparticles inhibit the virus from binding to host cells, as demonstrated in vitro.

## Background

Nanotechnology provides the ability to engineer the properties of materials by controlling their size, and this has driven research toward a multitude of potential uses for nanomaterials[1]. In the biological sciences, many applications for metal nanoparticles are being explored, including biosensors[2], labels for cells and biomolecules[3], and cancer therapeutics[4].

It has been demonstrated that, in the case of noble-metal nanocrystals, the electromagnetic, optical and catalytic properties are highly influenced by shape and size [5-7]. This has driven the development of synthesis routes that allow a better control of morphology and size [8-13]. Noble-metal nanomaterials have been synthesized using a variety of methods, including hard-template[14], bio-reduction[9] and solution phase syntheses[8,10-13].

Among noble-metal nanomaterials, silver nanoparticles have received considerable attention due to their attractive physicochemical properties. The surface plasmon resonance and large effective scattering cross section of individual silver nanoparticles make them ideal candidates for molecular labeling[15], where phenomena such as surface enhance Raman scattering (SERS) can be exploited. In addition, the strong toxicity that silver exhibits in various chemical forms to a wide range of microorganisms is very well known [16-18], and silver nanoparticles have recently been shown to be a promising antimicrobial material[19].

For these reasons, and based upon our previous work regarding interactions of noble metal nanoparticles with biomolecules[20], we decided to study the interaction of silver nanoparticles with viruses. Herein, we present the first findings of our investigation, the discovery that silver nanoparticles undergo size-dependent interaction with HIV-1.

## Findings

### Characterization of the tested silver nanoparticle preparations

The physicochemical properties of nanoparticles are strongly dependent upon their interactions with capping agent molecules[21]. Indeed, the surface chemistry of the nanoparticles can modify their interactions with external systems. For this reason we tested silver nanoparticles with three markedly different surface chemistries: foamy carbon, poly (N-vinyl-2-pyrrolidone) (PVP), and bovine serum albumin (BSA).

**Figure 1:**
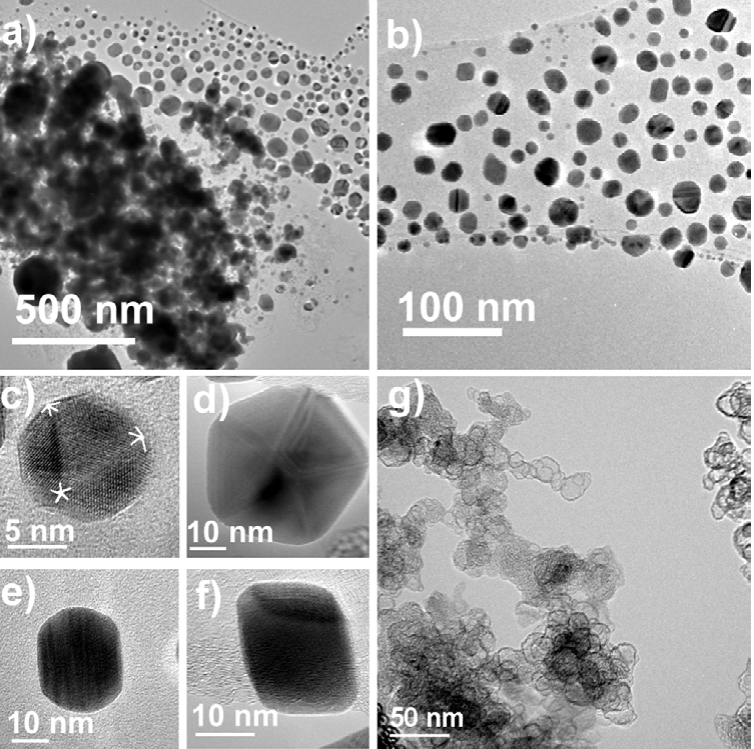
**Transmission electron microscopy (TEM) of the foamy carbon-coated silver nanoparticles. **a) TEM image of the sample prepared by dispersing the as-received powder in deionized water by ultra-sonication. The agglomeration of particles inside the foamy carbon matrix is observed. b) TEM image of nanoparticles outside of the carbon matrix. The broad distribution of shapes can be observed. c)-f) TEM images of nanoparticles with different morphologies. c) Icosahedral. d) Decahedral. e) Elongated. f) Octahedral. g) High Resolution TEM image of the foamy carbon matrix.

Foamy carbon-coated nanoparticles were obtained from Nanotechnologies, Inc., and used without further treatment. These nanoparticles are embedded in a foamy carbon matrix which prevents coalescence during their synthesis. The as-received nanoparticle sample consists of a fine black powder. For the purposes of the present work, the as-received powder was dispersed in deionized water by ultra-sonication. TEM analysis shows that the nanoparticles tend to be agglomerated inside the foamy carbon matrix, although a significant fraction of the population is released from this matrix by the energy provided from the ultra-sonic bath (Figure 1a–1f). These released nanoparticles are mainly free-surface nanoparticles, and it was observed that only nanoparticles that have escaped from the foamy carbon matrix interact with the HIV-1 cells.

**Figure 2:**
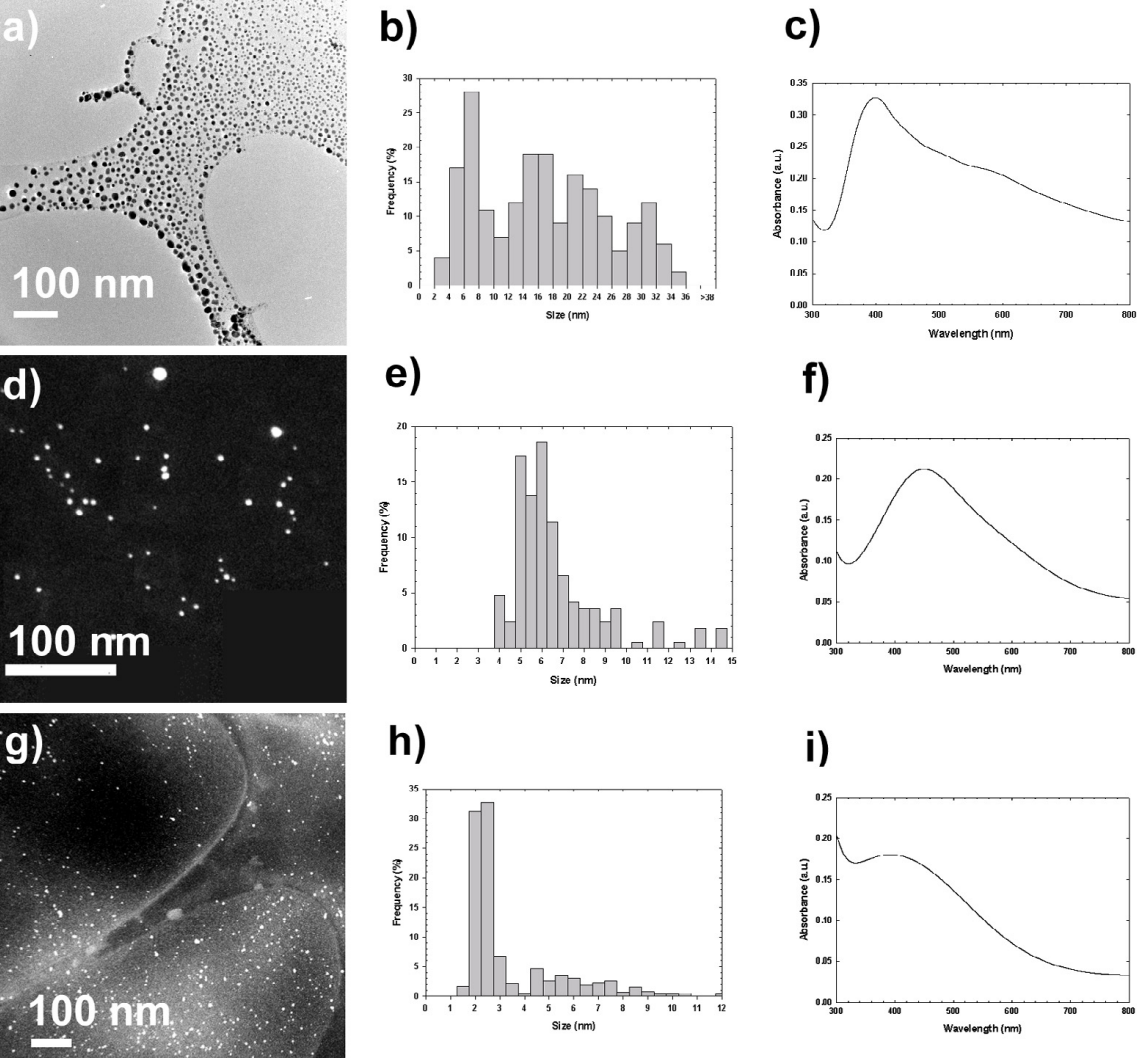
**Silver nanoparticle preparations. **a) TEM image of free surface silver nanoparticles released from the foamy carbon matrix by dispersing the as-received powder in deionized water by ultra-sonication. b) Size distribution of free surface nanoparticles measured by TEM analysis. c) UV-Visible spectrum of carbon-coated silver nanoparticles. d) HAADF image of PVP-coated silver nanoparticles. e) Size distribution of PVP-coated nanoparticles measured by TEM analysis. f) UV-Visible spectrum of PVP-coated silver nanoparticles. g) HAADF image of BSA-coated silver nanoparticles. h) Size distribution of BSA-coated nanoparticles measured by TEM analysis. i) UV-Visible spectrum of BSA-coated silver nanoparticles.

The interaction of the nanoparticles with the foamy carbon matrix is sufficiently weak that simply by condensing the TEM electron beam, even those nanoparticles that were not initially released by ultra-sonication are ejected from the foamy carbon agglomeration. In fact, after this experiment the complete size distribution of these nanoparticles is better observed, please refer to Additional file: 1. High resolution transmission electron microscopy (TEM) revealed that the silver nanoparticles released from the foamy carbon matrix by ultrasonication have a size distribution of 16.19 ± 8.68 nm (Figure 2a–b). By releasing the remaining nanoparticles from the foamy carbon matrix with the action of the electron beam, the average size was ~21 ± 18 nm. Additionally, TEM examination demonstrated that the sample is composed of several morphologies including multi-twinned nanoparticles with five-fold symmetry, i.e. decahedra and icosahedra, truncated pyramids, octahedral and cuboctahedral nanoparticles, among others (Figure 1c–1f).

PVP-coated nanoparticles were synthesized by the polyol method using glycerine as both reducing agent and solvent. In this method, a metal precursor is dissolved in a liquid polyol in the presence of a capping agent such as PVP[22]. PVP is a linear polymer and stabilizes the nanoparticle surface via bonding with the pyrrolidone ring. Infrared (IR) and X-ray photoelectron spectroscopy (XPS) studies have revealed that both oxygen and nitrogen atoms of the pyrrolidone ring can promote the adsorption of PVP chains onto the surface of silver[23]. The sample size distribution was obtained from high angle annular dark field (HAADF) images. The nanoparticles exhibited an average size of 6.53 nm with a standard deviation of 2.41 nm. (Figure 2d–e)

Silver nanoparticles directly conjugated to BSA protein molecules were synthesized in aqueous solution. Serum albumin is a globular protein, and is the most-abundant protein in blood plasma. Bovine serum albumin (BSA) is a single polypeptide chain composed of 583 amino acid residues [24]. Several residues of BSA have sulfur-, oxygen-, and nitrogen-bearing groups that can stabilize the nanoparticle surface. The strongest interactions with silver likely involve the 35 thiol-bearing cysteine residues. By using sodium borohydride, a strong reducing agent, BSA stabilizes nanoparticles via direct bonding with these thiol-bearing cysteine residues, and provides steric protection due to the bulkiness of the protein. The sample size distribution was obtained from HAADF images. Nearly 75% of the BSA-conjugated silver nanoparticles were 2.08 ± 0.42 nm in diameter, but a substantial fraction of larger particles was also observed, bringing the total average to 3.12 ± 2.00 nm (Figure 2g–h).

UV-visible spectroscopy is a valuable tool for structural characterization of silver nanoparticles. It is well known that the optical absorption spectra of metal nanoparticles are dominated by surface plasmon resonances (SPR), which shift to longer wavelengths with increasing particle size [25]. Also, it is well recognized that the absorbance of silver nanoparticles depends mainly upon size and shape [26,27]. In general, the number of SPR peaks decrease as the symmetry of the nanoparticle increases [27]. Recently, Schultz and coworkers[28] correlated the absorption spectra of individual silver nanoparticles with their size (40–120 nm) and shape (spheres, decahedrons, triangular truncated pyramids and platelets) determined by TEM. They found that spherical and roughly spherical nanoparticles, decahedral or pentagonal nanoparticles, and triangular truncated pyramids and platelets absorb in the blue, green and red part of the spectrum, respectively. In all the cases the SPR peak wavelength increases with size, as expected.

The UV-Visible spectra for all the nanoparticle preparations are shown in Figure 2. All samples presented a minimum at ~320 nm that corresponds to the wavelength at which the real and imaginary parts of the dielectric function of silver almost vanish [27]. The sample with carbon-coated silver nanoparticles exhibits four peaks at ~400, ~490, ~560 and ~680 nm, as shown in Figure 2c. The optical signature of this sample can be better understood in terms of the distribution of sizes and shapes observed in the TEM. As we previously mentioned, the distribution of shapes in the sample is broad, and a significant amount of nanoparticles are not spherical such as multi-twinned with five-fold symmetries. The presence of nanoparticles with pentagonal and triangular cross-sections could be responsible for the absorption at longer wavelengths. Thus, it is clear that the characteristic absorption of these nanoparticles arises from the contribution of different shapes and sizes, which agrees with the TEM observations.

On the other hand, the PVP-coated and BSA-coated silver nanoparticles present only one peak at ~450 and ~390 nm, respectively. These results indicate that both preparations are mainly composed by small spherical silver nanoparticles. It is also well know that for small particles a broadening of the plasmon absorption bands is expected, since there is a linear dependence of the full-width at half maximum (FWHM) with the reciprocal of the particle diameter[29]. The results for BSA-coated nanoparticles agree with the last statement, presenting just one broad symmetric peak at ~390 nm. On the other hand, the spectrum for the PVP-coated silver nanoparticles is not symmetric around the maximum absorption wavelength. In fact, this spectrum can be deconvoluted in two different curves, one centered at ~430 and another one at ~520 nm. The peak at ~430 nm could be assigned to the out-of-plane dipole resonance of the silver nanoparticles indicating the presence of spherical particles with small diameters. In addition, the synthesis of silver nanoparticles by the polyol method in presence of PVP promotes also the formation of multi-twinned nanoparticles (MTPs), being decahedra nanoparticles the most thermodynamically stable MTPs [23]. Therefore, the observed read shift is a consequence of both nanoparticles of larger size and the presence of decahedral nanoparticles with pentagonal cross sections.

### Interaction with HIV-1

High angle annular dark field (HAADF) scanning transmission electron microscopy was employed to study the interaction of silver nanoparticles with HIV-1. In our previous works, HAADF has proven to be a powerful technique for analysis of biological samples, such as proteins[20] and bacteria[30], interfaced with inorganic nanoparticles. HAADF images are primarily formed by electrons that have undergone Rutherford backscattering. As a result, image contrast is related to differences in atomic number [31] with intensity varying as ~Z^2^. Therefore, image contrast is strongly related to composition. As a good approximation, lighter elements appear dark and heavier elements appear bright. Due to a large difference in atomic number, silver nanoparticles are easily distinguished from the organic matter that composes the virus.

**Figure 3:**
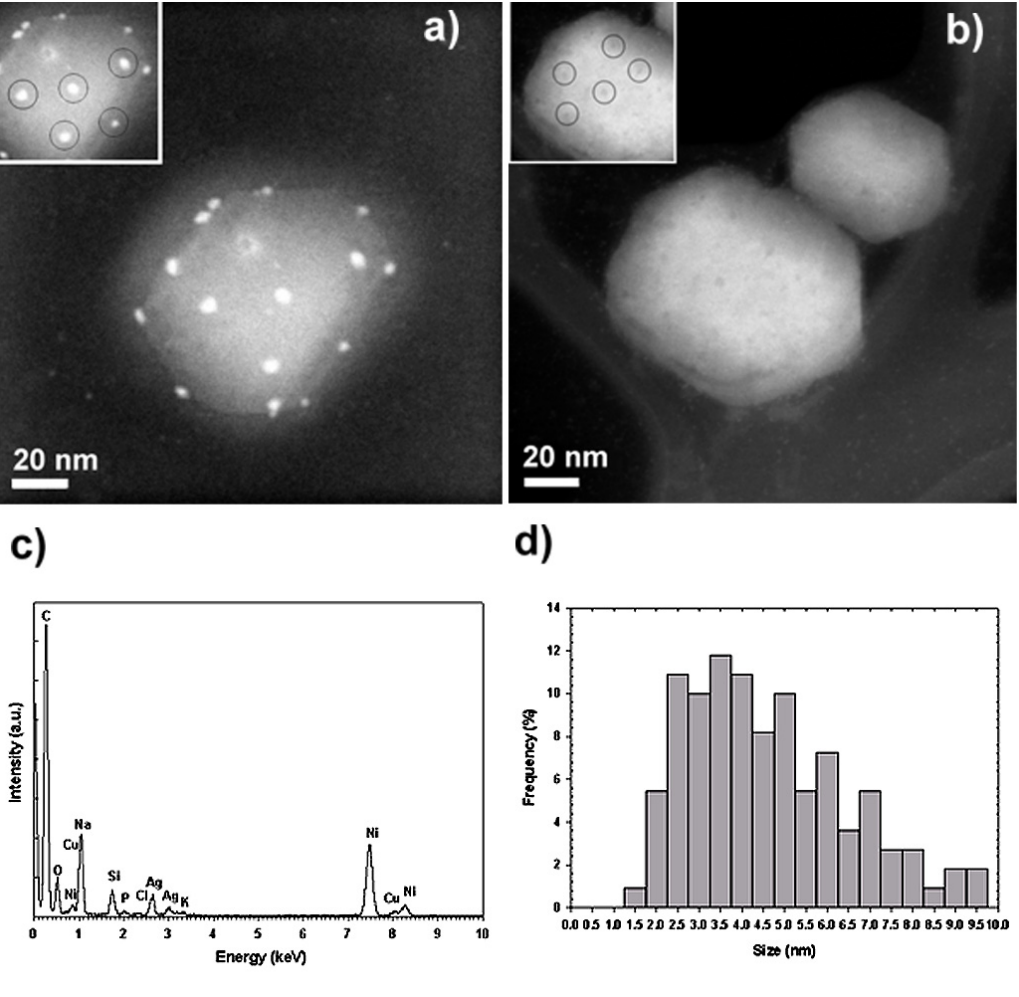
**HAADF images of the HIV-1 virus. **a) HAADF image of an HIV-1 virus exposed to BSA-conjugated silver nanoparticles. Inset shows the regular spatial arrangement between groups of three nanoparticles. b) HAADF image of HIV-1 viruses without silver nanoparticle treatment. Inset highlight the regular spatial arrangement observed on the surface of the untreated HIV-1 virus. c) EDS analysis of image a) confirming the presence of Ag. The C signal comes from both the TEM grid and the virus, O, and P are from the virus, and Na, Cl, and K are present in the culture medium. Ni and Si come from the TEM grid, while Cu is attributed to the sample holder. d) Composite size distribution of silver nanoparticles bound to the HIV-1 virus, derived from all tested preparations.

In Figure 3, we present HAADF images of the HIV-1 virus with (3a) and without (3b) silver nanoparticles. For complete experimental details, refer to Methods Section. The presence of silver was independently confirmed by Energy Dispersive X-ray Spectroscopy (EDS), shown in Figure 3c. Interestingly, the sizes of nanoparticles bound to the virus (Figure 3d) were exclusively within the range of 1–10 nm. In the case of the silver nanoparticles released from the carbon matrix, the fact that no nanoparticles greater than 10 nm in diameter were observed to interact with the virus is significant, since the size of ~40% of the overall population is beyond this range. This provides strong evidence for the size-dependence of interaction.

Additionally, the nanoparticles seen in Figure 3a are not randomly attached to the virus, as regular spatial relationships are observed among groups of three particles. Both the spatial arrangement of nanoparticles and the size dependence of interaction can be explained in terms of the HIV-1 viral envelope, and can provide insight into the mode of interaction between the virus and nanoparticles.

The exterior of the HIV-1 virus is comprised of a lipid membrane interspersed with protruding glycoprotein knobs, formed by trimers consisting of two subunits: the gp120 surface glycoprotein subunit is exposed to the exterior, and the gp41 transmembrane glycoprotein subunit spans the viral membrane and connects the exterior gp120 glycoprotein with the interior p17 matrix protein[32]. The main function of these protruding gp120 glycoprotein knobs is to bind with CD4 receptor sites on host cells. Numerous cellular proteins are also embedded within the viral envelope[33]. However, the protruding gp120 glycoprotein knobs are more exposed to the exterior, and should be more accessible for potential nanoparticle interactions.

Leonard and coworkers[34] reported that the gp120 subunit has nine disulfide bonds, three of which are located in the vicinity of the CD4 binding domain. These exposed disulfide bonds would be the most attractive sites for nanoparticles to interact with the virus. As mentioned previously, the nanoparticles in Figure 1a appear to be located at specific positions, with regular spatial relationships observed among groups of three particles. The observed spatial arrangements correlate with the positions of the gp120 glycoprotein knobs in the structural model for HIV-1 proposed by Nermut and coworkers[32].

Regular spatial relationships are also found on the surface of the untreated virus, as seen in the inset of Figure 1b. The observed darker contrast at these sites could indicate the locations of the glycoprotein knobs. As mentioned previously, contrast in HAADF images is strongly dependent on differences in atomic number. However, this is not the only factor in determining the image contrast. If the material is composed of elements of similar atomic numbers, as is the case for the organic constituents of the pure virus, local variations in sample density will provide noticeable contrast. The majority of the viral envelope consists of a densely-packed lipid membrane. However, for the glycoprotein knobs, we would expect a localized region of lower density due to the presence of membrane-spanning gp41 glycoproteins rather than the densely-packed lipids. Hence, these areas should appear darker than the rest of the viral envelope.

It has previously been determined that the center-to-center spacing between glycoprotein knobs is ~22 nm[35]. In the inset of Figure 3a, the average measured center-to-center spacing between silver nanoparticles is ~28 nm, which correlates with the expected spacing between glycoprotein knobs. The average center-to-center spacing between the small darker regions on the untreated virus is ~22 nm, which again suggests these sites are the gp120 glycoprotein knobs. Thus, the observed spatial arrangement of nanoparticles, the center-to-center distance between nanoparticles, and the fact that the exposed sulfur-bearing residues of the glycoprotein knobs would be attractive sites for nanoparticle interaction strongly suggest that silver nanoparticles interact with the HIV-1 virus via preferential binding to the gp120 glycoprotein knobs.

Presuming that the most attractive sites for interaction are the sulfur-bearing residues of the gp120 glycoprotein knobs, there are only a limited number of bonds that a nanoparticle can form. This limited number of stabilizing sites can explain why larger nanoparticles are not observed to attach to the virus. Assuming that each nanoparticle interacts with a single glycoprotein knob, and that each nearest-neighbor knob is occupied by another nanoparticle, from geometric considerations the theoretical upper limit for the diameter for these nanoparticles would be ~20 nm. However, if a nanoparticle larger than the diameter of one knob (~14 nm[35]) were to be attached, only a small fraction of the total nanoparticle surface would be anchored, resulting in a less stable interaction. Thus, if the nanoparticles are interacting with HIV-1 via preferential binding at gp120 glycoprotein knobs, we would expect to find mostly nanoparticles less than 14 nm in diameter, as particles in this size range would have the most stable surface interactions. This corresponds closely with our experimental observation that particles greater than 10 nm were not attached to the viral envelope.

Although the mechanism by which HIV infects host cells is not yet fully understood, there are two steps that are broadly agreed to be critical. The first step involves binding of gp120 to the CD4 receptor site on the host cell. Then, upon binding to CD4, a conformational change is induced in gp120, resulting in exposure of new binding sites for a chemokine receptor, i.e. CCR5 or CXCR4 [36-38]. An agent that preferentially interacts with the gp120 glycoprotein would block the virus from binding with host cells. Therefore, we measured the inhibitory effects of silver nanoparticles against HIV-1 in vitro.

**Figure 4:**
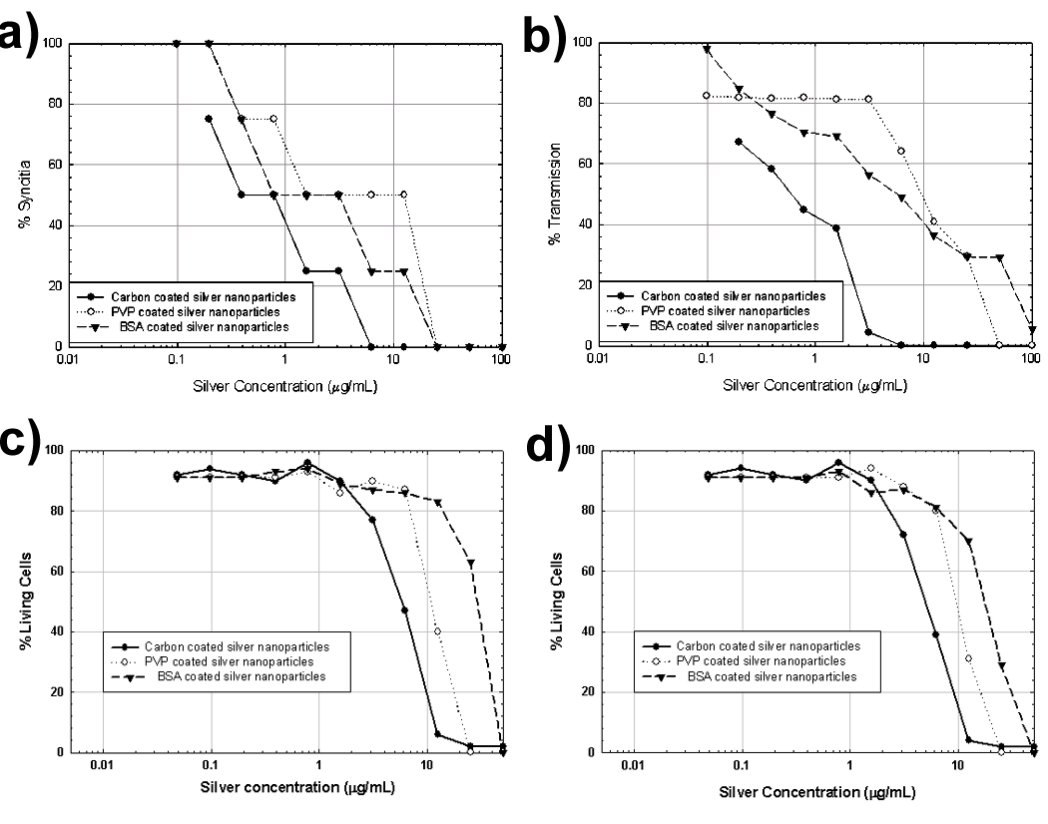
**Inhibition of HIV-1 and toxicity data. **a) Assessment of HIV-1 mediated syncytium formation in MT-2 cells. b) Percentage of HIV-1 transmission in cMAGI cells. The toxicity of the nanoparticle preparations against MT-2 cells was determined using the Trypan Blue exclusion assay. The samples were incubated at 37°C, and the cells were evaluated via optical microscopy after c) 3 h and d) 24 h of exposure to silver nanoparticles.

The capacity of silver nanoparticles to inhibit HIV-1 infectivity was determined by testing against CD4+ MT-2 cells and cMAGI HIV-1 reporter cells. For complete experimental details, refer to Methods Section. The cytopathic effects of CD4+ MT-2 infection were analysed by optical microscopy assessment of syncytium formation as described elsewhere[39,40], as well as by the HIV-1 infection of cMAGI cells using the Blue Cell Assay[41,42]. The cytotoxicity of all the nanoparticle preparations against MT-2 cells was determined using the Trypan Blue exclusion assay [43]. For all three nanoparticle preparations, at silver concentrations above 25 μg/mL, viral infectivity was reduced to an extent that it could not be detected by syncytium formation, as shown graphically in Figure 4. For each nanoparticle preparation, we found a dose-dependant inhibition of HIV-1 infectivity, with an IC_50 _where only moderate cell toxicity was observed, as seen in Figure 4.

Although the findings regarding interaction with HIV-1 were congruent among nanoparticles with markedly different surface chemistry, the toxicity and inhibition results were not the same. The differences in the observed trends in HIV-1 inhibition can be explained in terms of the capping agents employed for each nanoparticle preparation. BSA- and PVP-protected nanoparticles exhibit slightly lower inhibition because the nanoparticle surface is directly bound to and encapsulated by the capping agent. In contrast, the silver nanoparticles released from the carbon matrix have a greater inhibitory effect due to their essentially free surface area. The fact that the carbon-coated nanoparticles present higher cytotoxicity can also be explained in terms of surface chemistry. Since a significant amount of these silver nanoparticles possess nearly free surfaces, they are able to interact stronger with the host cells, thus increasing their toxicity. Clearly, selection of capping agents will be crucial for future research on the interaction of nanoparticles with viruses, microorganisms, and more complex biosystems, and many more variables require further testing, including the long-term effects of the presence of nanoparticles, and the impact of traces of precursor molecules and reaction by-products.

In conclusion, we have found that silver nanoparticles undergo size-dependent interaction with HIV-1, and that the bound particles exhibit regular spatial relationships. These observations lead us to suggest that the nanoparticles undergo preferential binding with the gp120 subunit of the viral envelope glycoprotein. Silver nanoparticles inhibit the HIV-1 virus infectivity in vitro, which also supports our proposal regarding preferential interaction with gp120. These findings only provide indirect evidence for our proposed mode of interaction, and we are currently undertaking testing to determine conclusively if direct conjugation between gp120 and silver nanoparticles exist.

The interactions of inorganic nanoparticles with biosystems are just beginning to be understood, and potential applications are being discovered at an increasing rate. However, in order to realize the future promise of nanoscience, it is imperative that the toxicity and long-term health effects of exposure to nanomaterials be fully explored. The flexibility of nanoparticle preparation methods, the multitude of functionalization techniques, and facile incorporation of nanoparticles into a variety of media provide the incentive for further research on the interaction of metal nanoparticles with viruses.

## Methods

### a) HIV-1 strains and cell lines

HIV-1_IIIB _laboratory strain of HIV-1 an X4 wild type (wt) virus was obtained through the AIDS Research and Reference Reagent Program, Division of AIDS, NIAID, NIH. CD4+ MT-2 cell line was obtained from the American Type Culture Collection. The cMAGI HIV-1 reporter cells were a generous gift from Dr. Phalguni Gupta from the University of Pittsburgh. All other reagents used were of the highest quality available.

cMAGI cells were cultured in DMEM Dulbecco's Modified Eagle Medium (DMEM) (1X) liquid without sodium phosphate and sodium pyruvate. The medium contained 4,500 mg/L D-glucose and L-glutamine (Invitrogen, Paisley, UK), with 10% fetal calf serum (FCS), 0.2 mg/mL geneticin (G418), and 0.1 μg/mL puromycin. MT-2 cells were cultured in RPMI 1640, containing 10% fetal calf serum (FCS) and antibiotics.

HIV-1_IIIB _primary clinical isolates were propagated by subculture in MT-2 and cMAGI cells. HIV-1_IIIB _was reproduced according to the DAIDS Virology Manual for HIV Laboratories, version 1997, compiled by Division of AIDS of the National Institute of Allergies and Infectious Diseases and the National Institute of Health, and Collaborators. Aliquots of cell-free culture viral supernatants were used as viral inocula.

All the work related to HIV-1 cells, except for TEM analysis, was done in a Biosafety Level 3 (BSL-3) Laboratory.

### b) Synthesis of the three different silver nanoparticle preparations

Carbon coated silver nanoparticles tested in this study were obtained from Nanotechnologies, Inc. and used without further treatment. For more information about the synthesis of these nanoparticles, please visit 

PVP-coated silver nanoparticles were synthesized by the polyol method using glycerine as both reducing agent and solvent. Silver sulfate (Ag_2_SO_4_, reagent grade) and poly (N-vinyl-2-pyrrolidone) (PVP-K30, MW = 40,000) were purchased from Sigma Aldrich and 1,2,3-Propanetriol (Glycerin, >99%) was purchased from Fischer Chemicals, all the materials were used without any further treatment. Briefly, we added 0.2 g of PVP to a round bottom flask following by the addition of 30 mL of glycerin. Once PVP was dissolved, we increased the temperature to 140°C. After 30 minutes we added 2 mL of 0.015 M Ag_2_SO_4 _and left to react for 1 h.

Silver nanoparticles directly conjugated to bovine serum albumin (BSA) protein molecules were produced as following described. Silver nitrate (AgNO_3_, 0.945 M), sodium borohydride (NaBH_4_, 99%) and 200 proof spectrophotometric-grade ethanol were purchased from Aldrich. Bovine serum albumin (BSA) was purchased from Fisher and was used without further treatment. Briefly, sodium borohydride was added to an aqueous solution of silver nitrate and BSA under vigorous stirring. The molar ratio of Ag^+^:BSA was 28:1, and the molar ratio of Ag^+^:BH4^- ^was 1:1. The reaction volume was 40 mL, and contained 13.50 μmol BSA. The reaction was allowed to proceed for 1 h, and the product was purified by precipitation at -5°C, followed by cold ethanol filtration.

### c) Characterization of the different silver nanoparticle preparations

Transmission electron microscope was conducted in a HRTEM JEOL 2010F microscope equipped with Schottky-type field emission gun, ultra-high resolution pole piece (Cs = 0.5 mm), and a scanning transmission electron microscope (STEM) unit with high angle annular dark field (HAADF) detector operating at 200 kV. Briefly, a droplet of each different solution of silver nanoparticles was placed on a Cu grid with lacey carbon film (Ted Pella), and allowed to evaporate. Size distributions for each nanoparticle preparation were obtained from TEM analysis based on the measurement of 400 particles, and 600 particles in the case of BSA-coated nanoparticles.

UV-visible spectra were obtained at room temperature using a 10 mm path length quartz cuvette in a Cary 5000 spectrometer. All the solutions were diluted 30 × in deionized water before acquiring the spectra.

### d) Electron microscopy of HIV-1 and silver nanoparticles

Samples were prepared for electron microscopy as follows: 10^5 ^TCID_50 _samples of HIV-1_IIIB _cell free virus were treated with solutions of the different silver nanoparticles at a concentration of 100 μg/mL. After 30 seconds, a 10 μL droplet was deposit on a carbon coated nickel TEM grid and exposed to a 2.5% solution of PBS/glutaraldehyde vapors for 30 minutes. Microscopy was done using a JEOL 2010-F TEM equipped with an Oxford EDS unit, at an accelerating voltage of 200 kV and operated in scanning mode using an HAADF detector.

### e) Inhibition of HIV-1 with silver nanoparticles

RPMI medium only or containing varying concentrations of silver nanoparticles were mixed with samples 10^5 ^TCID_50 _of HIV-1_IIIB _cell free virus. The highest concentration of silver nanoparticles used was 100 μg/mL. After 30 seconds, sequential 2-fold dilutions of the solutions were added to cultures of target cells (2 × 10^5 ^MT-2 and 2 × 10^5 ^cMAGI HIV-1 reporter cells with 0.2–0.5 multiplicity of infection (m.o.i) of HIV-1_IIIB _virus) prepared as previously mentioned. Each dilution was exposed to four replicate wells. After that, the cells were incubated in a 5% CO_2 _humidified incubator at 37°C for 3–5 days. Assessment of HIV-1 mediated syncytium formation was performed for the MT-2 cells, while for cMAGI cells, the percentage of transmission was estimated as follows: the number of blue-stained cells obtained from the supernatant of each of the tested wells was divided by the number of blue-stained cells obtained from the culture supernatant in the well of the positive control.

### f) Cytotoxicity of silver nanoparticles against MT-2 cells

The cytotoxicity of the nanoparticle preparations against MT-2 cells was determined using the Trypan Blue exclusion assay. In all cases, the initial concentration of silver nanoparticles was 50 μg/mL and sequential two-fold dilutions were made and mixed with 2 × 10^5 ^MT-2 cells. The samples were incubated at 37°C, and the cells were evaluated via optical microscopy after 3 h and 24 h of exposure to silver nanoparticles. Briefly, an aliquot of the cell suspension was diluted 1:1 (v/v) with 0.4% Trypan Blue and the cells were counted using a haemocytometer. Viability was expressed as the percentage of number of unstained treated cells to that of the total number of cells.

## Supplementary Material

Additional File 1 Supporting information. The file is a word document that contains the complete size distribution of the carbon-coated silver nanoparticles evaluated by TEM Click here for file
