# Supplementary material for: Interaction of silver nanoparticles with HIV-1
Source: J Nanobiotechnology. 2005 Jun 29;3:6. doi: 10.1186/1477-3155-3-6 (PMC1190212; doi:10.1186/1477-3155-3-6)
Supplement: Additional File 1 — Supporting information. The file is a word document that contains the complete size distribution of the carbon-coated silver nanoparticles evaluated by TEM [file 1477-3155-3-6-S1.doc]

# Supporting Information

**a) Electron microscopy image and size distribution histogram of the carbon-coated silver nanoparticles:**


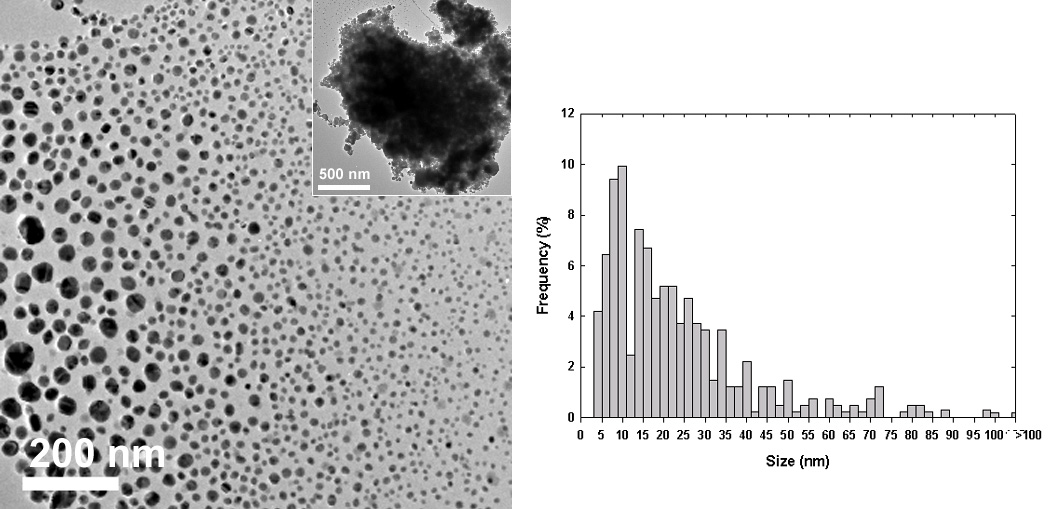


*Left:* The inset in the Figure shows the original agglomerate of particles that was dispersed by the action of the electron beam in the TEM, as described in the manuscript. *Right:* After, this experiment the complete size distribution of these nanoparticles is observed.
